# Nail Wound of the Foot

**Published:** 1891-06

**Authors:** A. W. Clement


### NAIL WOUND OF THE FOOT.

### SYNOVITIS, SEPTIC INFECTION, DEATH.

BY A. W. CLEMENT, V. S.

*Subject:* — A bay gelding seven years old used for carriage purposes.

CLINICAL HISTORY:—The animal stepped upon a nail in the street while being delivered to the buyer by a man in the employ of the seller. The shipper missed the boat and on his return was about three rods from the stable when the accident happened. The stable men could not remove the nail so called in a blacksmith. The blacksmith had considerable difficulty in getting the nail out. When removed it was found that the nail had passed into the foot to the depth of nearly two inches. From the way in which the nail was bent it had evidently passed upward and slightly backward. The nail entered the near hind foot by the side of the frog at about the beginning of the posterior third.

The blacksmith pared the foot down, and by direction of the seller a poultice of cow manure was applied. At the request of the buyer I visited the animal three days after the accident happened. I found the animal standing on three legs, apparently suffering great pain. Pulse 60 and very full. Temperature 101°. Respiration 22. The animal was eating well and had full control over the muscles of the jaw.

I removed the bandage and cleaned the foot. There was some swelling and considerable heat just above the hoof. After paring away more of the sole and frog, the wound at the base of the foot was exposed. A probe could be introduced into the wound to the depth of an inch. The parts were irrigated with corrosive sublimate solution 1 in 1000, then dressed with iodoform and several layers of wood-wool and absorbent cotton, and the whole enclosed in a moist crinolin bandage. The dressings were changed daily. The discharge of pus from the wound was very slight but there was a constant escape of synovia. The tissues became very œdematous, the swelling extending for some distance above the hock joint. The temperature on the second day was 103° and on the third day 106° at which point it remained until death. Abscesses formed just above the coronary band and, later, around the fetlock joint. Sinuses led from the abscesses above the coronary band to the coronary and pedal bones and finally became so large that one could easily pass his finger into the corono-pedal articulation. The animal lived for twelve days after the accident happened.

Post mortem. Examination of the foot. As it was impossible, for want of time, to make a full post mortem examination of the animal, the leg was sawn through midway of the large metalarsal bone and a vertical incision made through the foot and the part of the leg attached. It was seen that the nail had passed through the edge of the fatty frog, into the navicular joint, and through the navicular bone into the coffin joint. The edges of the opening through the navicular bone were quite irregular. There was intense reddening of the articular surfaces of the navicular, pedal and coronary bones. The articular surfaces were smooth, however. The primary wound communicated freely with the sinuses leading from the abscesses just above the coronary band.

